# Object recognition with hierarchical discriminant saliency networks

**DOI:** 10.3389/fncom.2014.00109

**Published:** 2014-09-09

**Authors:** Sunhyoung Han, Nuno Vasconcelos

**Affiliations:** ^1^Analytics Department, ID AnalyticsSan Diego, CA, USA; ^2^Statistical and Visual Computing Lab, Electrical and Computer Engineering, University of CaliforniaSan Diego, La Jolla, CA, USA

**Keywords:** object recognition, object detection, top-down saliency, discriminant saliency, hierarchical network

## Abstract

The benefits of integrating attention and object recognition are investigated. While attention is frequently modeled as a pre-processor for recognition, we investigate the hypothesis that attention is an intrinsic component of recognition and vice-versa. This hypothesis is tested with a recognition model, the hierarchical discriminant saliency network (HDSN), whose layers are top-down saliency detectors, tuned for a visual class according to the principles of discriminant saliency. As a model of neural computation, the HDSN has two possible implementations. In a biologically plausible implementation, all layers comply with the standard neurophysiological model of visual cortex, with sub-layers of simple and complex units that implement a combination of filtering, divisive normalization, pooling, and non-linearities. In a convolutional neural network implementation, all layers are convolutional and implement a combination of filtering, rectification, and pooling. The rectification is performed with a parametric extension of the now popular rectified linear units (ReLUs), whose parameters can be tuned for the detection of target object classes. This enables a number of functional enhancements over neural network models that lack a connection to saliency, including optimal feature denoising mechanisms for recognition, modulation of saliency responses by the discriminant power of the underlying features, and the ability to detect both feature presence and absence. In either implementation, each layer has a precise statistical interpretation, and all parameters are tuned by statistical learning. Each saliency detection layer learns more discriminant saliency templates than its predecessors and higher layers have larger pooling fields. This enables the HDSN to simultaneously achieve high selectivity to target object classes and invariance. The performance of the network in saliency and object recognition tasks is compared to those of models from the biological and computer vision literatures. This demonstrates benefits for all the functional enhancements of the HDSN, the class tuning inherent to discriminant saliency, and saliency layers based on templates of increasing target selectivity and invariance. Altogether, these experiments suggest that there are non-trivial benefits in integrating attention and recognition.

## 1. Introduction

Recent research in computational neuroscience has enabled significant advances in the modeling of object recognition in visual cortex. These advances are encoded in recent object recognition models, such as HMAX (Riesenhuber and Poggio, 1999; Serre et al., 2007; Mutch and Lowe, 2008) the convolutional networks of Pinto et al. (2008); Jarrett et al. (2009) and a number of deep learning models (Hinton et al., 2006; Krizhevsky et al., 2012). When compared to classical sigmoid networks (LeCun et al., 1990, 1998), these models reflect an improved understanding of the neurophysiology of visual cortex (Graham, 2011), recently summarized by the standard neurophysiological model of Carandini et al. (2005). This consists of hierarchical layers of simple and complex cells (Hubel and Wiesel, 1962). Simple cells implement a combination of filtering, rectification, divisive contrast normalization, and sigmoidal non-linearity, which makes them *selective* to certain visual features, e.g., orientation. Complex cells pool information from multiple simple cells, producing an *invariant* representation. While the receptive fields of cells at the lower hierarchical levels resemble Gabor filters of limited spatial extent, cells at the higher layers have much more complex receptive fields, and pool information from larger regions of support (Poggio and Edelman, 1990; Perrett and Oram, 1993). This makes them more *selective* and *invariant* than their low-level counterparts. Extensive experiments have shown that accounting for simple and complex cells (Serre et al., 2007), using normalization and rectification (Jarrett et al., 2009), optimizing the sequence of these operations (Pinto et al., 2009), or learning deep networks with multiple layers (Krizhevsky et al., 2012) can be highly beneficial in terms of recognition performance.

There are, nevertheless many aspects of cortical processing that remain poorly understood. In this work, we consider the role of attention in object recognition, namely how attention and recognition can be integrated in a shared computational architecture. We consider, in particular, the saliency circuits that drive the attention system. These circuits are usually classified as either bottom-up or top-down. Bottom-up mechanisms are stimulus driven, driving attention to image regions of conspicuous stimuli. Many computational models of bottom-up saliency have been proposed in the literature. They equate saliency to center-surround operations (Itti et al., 1998; Gao and Vasconcelos, 2009), frequency analysis (Hou and Zhang, 2007; Guo et al., 2008), or stimuli with specific properties, e.g., low-probability (Rosenholtz, 1999; Bruce and Tsotsos, 2006; Zhang et al., 2008), high entropy (Kadir and Brady, 2001), or high complexity (Sebe and Lew, 2003). An extensive review of bottom-saliency models is available in Borji and Itti (2013) and an experimental comparison of their ability to predict human eye fixations in Borji et al. (2013). While these mechanisms can speed up object recognition (Miau and Schmid, 2001; Walther and Koch, 2006), by avoiding an exhaustive scan of the visual scene, they are not intrinsically connected to any recognition task. Instead, bottom-up saliency is mostly a pre-processor of the visual stimulus, driving attention to regions that are likely to be of general vision interest. On the other hand, top-down saliency mechanism are task-dependent, and emphasize the visual features that are most informative for a given visual task. These mechanisms assign different degrees of saliency to different components of a scene, depending on the recognition task to be performed. For example, it is well known since the early studies of Yarbus (1967) that, when subjects are asked to search for different objects in a scene, their eye fixation patterns can vary significantly. It has also long been known that attention has a feature based component. More precisely, human saliency judgments can be manipulated by enhancement or inhibition of the feature channels of early vision, e.g., color or orientation (Maunsell and Treue, 2006). This type of feature selection should, in principle, be useful for recognition.

Overall, there are several reasons to study the integration of recognition and top-down saliency. First, the ability to simultaneously achieve selectivity and invariance is a critical requirement of robust image representations for recognition. By increasing the selectivity of neural circuits to certain classes of stimuli, the addition of top-down saliency, which increases selectivity to the object classes of interest, could potentially improve recognition performance. Second, there is some evidence that adding an attention mechanism to computational models of object recognition can improve their performance. For example, spatially selective units are known to substantially improve HMAX performance (Mutch and Lowe, 2008). In fact, as we will show later in this work, some of the recent object recognition advances in computer vision, such as the now widely used SIFT descriptor, can be interpreted as saliency mechanisms. Although these are purely stimulus driven, i.e., bottom-up, the gains with which they are credited again suggest that saliency has a role to play in recognition. Third, the connection to saliency provides the intermediate layers of a recognition network with a functional justification. Rather than a side effect of a holistic network optimization with respect to a global recognition criterion, they become individual saliency detectors, each attempting to improve on the saliency detection performance of their predecessors. This has a simpler evolutionary justification, under which (1) visual systems would evolve one layer at a time and (2) the search for improved performance in attention tasks leads naturally to object recognition networks.

All these observations suggest the hypothesis that, rather than a simple bottom-up pre-processor that determines conspicuous locations to be sequentially analyzed by the visual system, saliency could be embedded in object recognition circuits. Our previous work has also shown that, under the discriminant saliency principle, the computations of saliency can be mapped to the standard neurophysiological model (Gao et al., 2008; Gao and Vasconcelos, 2009). While we have exploited this mapping extensively for modeling bottom-up saliency, the underlying computations can be naturally extended to top-down saliency. In fact, under this extension, the saliency operation boils down to the discrimination between an object class and the class of natural images. This is intrinsically connected to object recognition. It, thus, appears that biology could have chosen to embed saliency in the recognition circuitry, if this had an evolutionary benefit, i.e., if embedding saliency in object recognition networks improves recognition performance. One of the goals of this work is to investigate this question. For this, we propose a family of *hierarchical discriminant saliency networks (HDSNs)*, which jointly implement attention and recognition. More precisely, HDSNs are networks whose layers implement top-down saliency detection, based on features of increasing selectivity and invariance. These layers are stacked, so as to enhance the saliency detection of their predecessors. Since higher layers become more selective for the target objects, object recognition should be enhanced as a by-product of the saliency computation. All saliency detectors are derived from the discriminant saliency principle of Gao and Vasconcelos (2009) and explicitly minimize recognition error, using the top-down saliency measure of Gao et al. (2009). This is implemented with the biologically plausible computations of Gao and Vasconcelos (2009). In this way, HDSNs are consistent with the standard neurophysiological model (Carandini et al., 2005), but have a precise computational justification, and a statistical interpretation for all network computations. All parameters can thus be tuned by statistical learning, enabling the explicit optimization of the network for recognition.

A number of properties of HDSNs are investigated in this work. We start by showing that HSDNs can be implemented in multiple ways. In addition to the biologically plausible implementation, they can be interpreted as an extension of convolutional neural network models commonly used for recognition. This extension consists of a new type of rectifier function, which is a generalization of the recently popular rectified linear unit (ReLU) (Nair and Hinton, 2010; Krizhevsky et al., 2012). The generalization is parametric and can be tuned according to the statistics of the object classes of interest. This tuning enables the network to implement behaviors, such as switching from selectivity to feature presence to selectivity to feature absence, that are not possible with the units in common use. The computation of saliency also enables the network to learn more discriminant receptive fields. In result, receptive fields at the higher network layers become tuned for configurations of salient low-level features, improving both saliency and object recognition performance. Overall, HDSNs are shown to exhibit the ability to model both salient features and their configurations, to replicate the human ability to identify objects due to both feature presence and absence, to modulate saliency responses according to the discriminant power of the underlying features, and to implement optimal feature denoising for recognition. The introduction of HDSNs is complemented by the analysis of several recognition methods from computer vision (Vasconcelos and Lippman, 2000; Lazebnik et al., 2006; Zhang et al., 2007; Boiman et al., 2008; Yang et al., 2009; Zhou et al., 2009), which are mapped to a canonical architecture with many of the attributes of the biological models. This enables a clear comparison of methods from the two literatures. A rigorously controlled investigation, involving models from both computational neuroscience and computer vision, shows that there are recognition benefits to both the class-tuning inherent to discriminant saliency and the hierarchical organization of the HDSN into saliency layers of increased target selectivity and invariance. Experiments on standard visual recognition datasets, as well as a challenging dataset for saliency, involving the detection of panda bears in a cluttered habitat, show that these advantages can translate into significant gains for object detection, localization, recognition, and scene classification.

## 2. Methods

We start with a brief review of discriminant saliency.

### 2.1. Discriminant saliency

Discriminant saliency is derived from two main principles: that (1) neurons are optimal decision-making devices and (2) optimality is tuned to the statistics of natural visual stimuli. The visual stimulus is first projected into the receptive field of a neuron, through a *linear transformation* 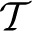, which produces a *feature response X*. The neuron then attempts to classify the stimulus as either belonging to a *target* or *background* (also denoted *null*) class. The definitions of target and background class define the saliency operation. For bottom-up saliency, they are the feature responses in a pair of center (target) and surround (background) windows co-located with the receptive field (Gao et al., 2008; Gao and Vasconcelos, 2009). In this work we consider the problem of top-down saliency, where the target class is defined by the feature responses to a stimulus class of interest and the background class by the feature responses to the class of natural images (Gao et al., 2009). In the object recognition context, the stimulus class of interest is a class of objects. Neurons implement the optimal decision rule for stimulus classification in the minimum probability of error (MPE) sense (Duda et al., 2001; Vasconcelos, 2004a). Saliency is then formulated as the discriminability of the visual stimulus with respect to this classification. Stimuli that can be easily assigned to the target class are denoted salient, otherwise they are not salient. The discriminability score used to measure stimulus saliency is computed in two steps, implemented by two classes of neurons that comply with the classical grouping into simple and complex cells. Simple cells first compute the optimal decision rule for stimulus classification into target and background, at each location of the visual field. Complex cells then combine simple cell outputs to produce a discriminability score.

#### 2.1.1. Statistical model

Consider a simple cell, whose receptive field is centered at location *l* of the visual field. The visual stimulus at *l* is drawn from class *Y*(*l*), where *Y*(*l*) = 1 for target and *Y*(*l*) = 0 for background. The goal of the cell is to determine *Y*(*l*). For this, it applies a linear transformation 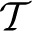 to the stimulus in a neighborhood of *l* (the receptive field of the cell), producing a feature response *X*(*l*) at that location. The details of the transformation are not critical, the only constraint is that it is a bandpass transformation. Using the well know-fact that bandpass feature responses to natural images follow the generalized Gaussian distribution (GGD) (Buccigrossi and Simoncelli, 1999; Huang and Mumford, 1999; Do and Vetterli, 2002), the feature distributions for target and background are

(1) $$P_{X|Y}(x(l)|i)=\frac{\beta }{2\alpha \Gamma (1/\beta )}e^{-{\left(\frac{\left|x(l)\right|}{\alpha_{i}}\right)}^{\beta }}\;i\in \{0,1\}.$$

The parameters α_*i*_ are the *scales* (variances) of the two distributions, while β is a parameter that determines their shape. For natural imagery, β is remarkably consistent, taking values around 0.5 (Srivastava et al., 2003). This value is assumed in the remainder of this work. The scales α_*i*_ are learned from two training samples *R*_1_, *R*_*o*_ of examples from target and null class, respectively, by maximum a posteriori (MAP) estimation, using a conjugate Gamma prior of hyper-parameters η, ν. As described in Gao and Vasconcelos (2009) the MAP estimates of α_1_ and α_0_ are

(2) $$\alpha_{i}^{\beta }=\frac{1}{\kappa }\sum_{x_{j}\in R_{i}}|x_{j}{|}^{\beta }+\nu ,\;i\in \{0,1\},\;\kappa =\frac{n+\eta }{\beta }.$$

The values of the prior parameters are not critical. They are used mostly to guarantee that the estimates of α_*i*_ are non-zero. In this work, we use η = 1 and ν = 10^−3^.

#### 2.1.2. Saliency measure

**Figure 1:**
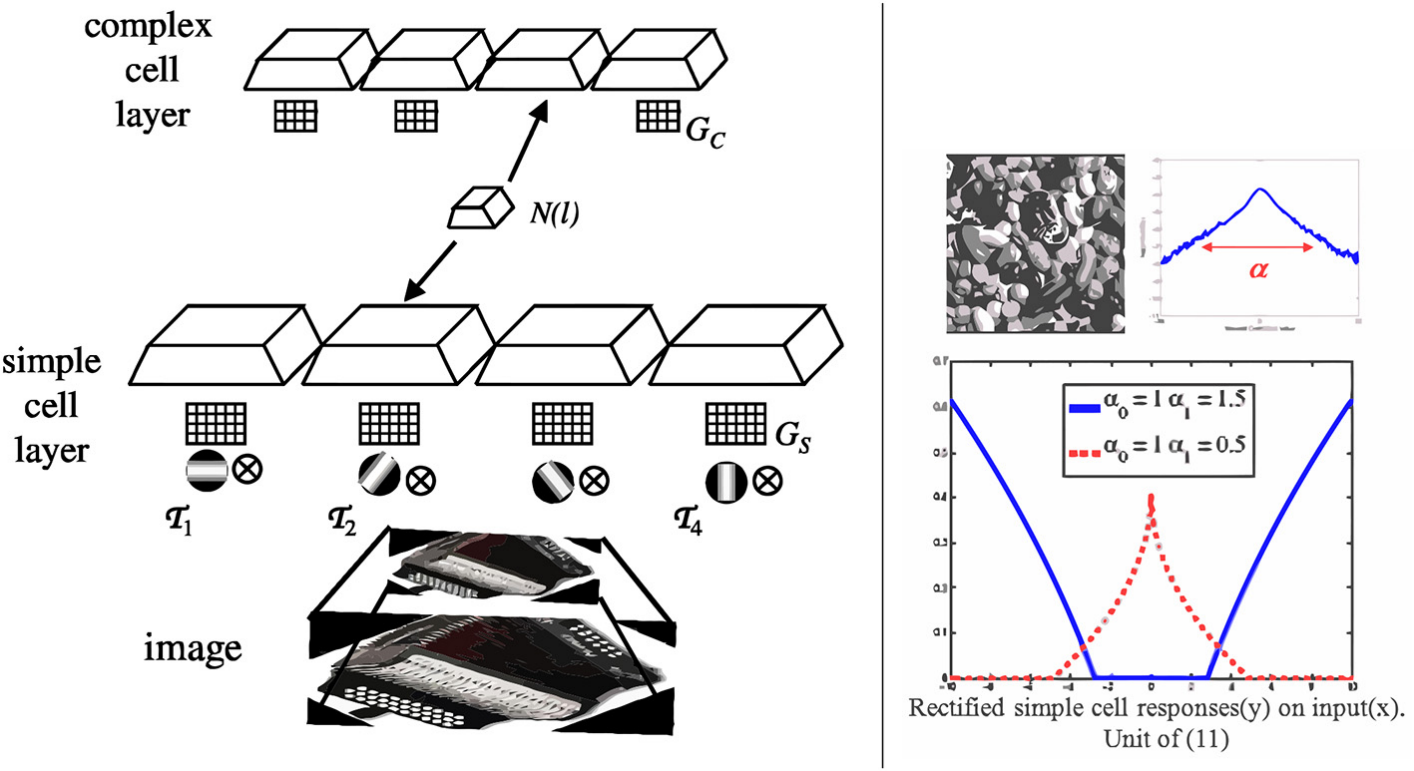
**Left:** saliency is computed by a pair of layers of simple and complex cells. In the simple cell layer, the visual stimulus is first subject to a number of linear transformations, which are repeated at various image scales, illustrated by chopped pyramids. In the example of the figure, the set of transformations consist of four oriented filters 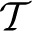
_*i*_. Each simple cell computes the optimal decision rule for the classification of the filter response at one scale and location of a simple cell grid *G_S_*. A channel consists of all retinotopic maps of simple cell response derived from a common transformation (4 channels in the figure). A complex cell computes the saliency score of (7), using a pooling neighborhood *N*(*l*) that spans locations and scales. The retinotopic maps of complex cell response are in one to one correspondence to those of simple cell response, but the grid *G_C_* of complex cell locations is a subsampled replica of its simple cell counterpart. The simple and complex cell computations can be implemented in two ways. In a biologically plausible implementation, simple cells compute the posterior probabilities of (3), while complex cells implement the pooling operator of (8). In an artificial neural network implementation, simple cells implement the parametric ReLU units of (11), while complex cells perform simple averaging. **Right**: the top inset shows the histogram of responses of a bandpass filter to the natural image on the left. The scale parameter α characterizes the spread of the distribution and is large (small) for filters that match (do not match) structures in the image, i.e., features that are “present” (“absent”). The plot in the bottom shows the function of (11) for different values of α_*i*_. The behavior of the parametric ReLU can change from the detection of feature presence to the detection of feature absence, depending on the scales of the target and background GGD distributions. The curve in red (blue) corresponds to a feature present (absent) in the target but absent (present) in the null class.

A simple cell uses the above model of natural image statistics to compute the posterior probability of the target class, given the observed feature response *X*(*l*)

(3) $$P_{Y|X}\left(1|x(l)\right)=\sigma \left(g[x(l)]\right),$$

where σ(*x*) = 1/(1 + *e*^−*x*^) is the sigmoid function and *g*(*x*) the log-likelihood ratio (LLR)

(4) $$g(x)=\log\frac{P_{X|Y}(x|1)}{P_{X|Y}(x|0)}={\left(\frac{\left|x\right|}{\alpha_{0}}\right)}^{\beta }-{\left(\frac{\left|x\right|}{\alpha_{1}}\right)}^{\beta }+T,$$

with $T=\log\left(\frac{\alpha_{0}}{\alpha_{1}}\right)$. Simple cells are organized into convolutional layers, which repeat the simple cell computation at each location of the visual field. Each layer produces a retinotopic map of posterior probabilities *P*_*Y*|*X*_(1|*x*(*l*)) given the feature responses derived from a common transformation 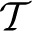. The computation is repeated for various transformations 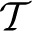
_*i*_, producing several *channels* of simple cell response. As illustrated in the left of Figure 1, these channels are computed at multiple resolutions, by applying each transformation to re-scaled replicas of the visual stimulus. In our implementation, we use 10 scales, with subsampling factors of 2^*i*/4^, *i* ∈ {0, …, 9}.

The saliency of the stimulus at location *l* is evaluated by a complex cell that combines the responses of afferent simple cell responses in a neighborhood *N*(*l*) (its pooling neighborhood) into the discriminability score

(5) $$S(l)=E_{X(l)}\left({\left\lfloor g(X)\right\rfloor }_{+}\right),$$

where *E*_*X*(*l*)_ denotes the expectation with respect to the distribution of *X* in *N*(*l*) and ⌊*x*⌋_+_ = max(*x*, 0) is the half-wave rectification function. This rectification assures that the score is non-negative, by zeroing the LLR *g*(*x*) at all locations where the outcome of the Bayes decision rule for MPE classification

(6) $$\hat{Y}(l)=\;\{\begin{array}{ll} 1, & if\;g[x(l)]\geq 0\\ 0, & if\;g[x(l)]<0 \end{array}$$

assigns the response to the background class (i.e., chooses Ŷ(*l*) = 0). Large values of the score *S*(*l*) indicate that the feature response *X*(*l*) can be clearly assigned to the target class, i.e., the LLR *g*(*x*) is both positive and large. For such stimuli, the posterior probability of (3) is close to one. In this case, the visual stimulus is salient. Small scores indicate that this is not the case. The computation of the saliency score of (5) is implemented by replacing the expectation with a sample average over *N*(*l*)

(7) $$S(l)=\frac{1}{\left|N(l)\right|}\sum_{j\;\in \;N(l)}\lfloor g[x(j)]\rfloor_{+}.$$

This is computed as a combination of the responses of simple cells in *N*(*l*), since (7) can be written as Han and Vasconcelos (2010)

(8) $$S(l)\;=\frac{1}{\left|N(l)\right|}\sum_{j\in N(l)}\xi \{P_{Y|X}[1|x(j)]\}$$

with

$$\xi (x)=\{\begin{array}{ll} \frac{1}{2}\log\frac{x}{1-x}, & x\geq .5\\ 0, & x<.5. \end{array}$$

Hence, a complex cell applies the non-linear transformation ξ(*x*) to the responses of the afferent simple cells and pools the transformed responses into the saliency measure *S*(*l*). The neighborhood *N*(*l*) is thus denoted as the *pooling neighborhood* of the complex cell. Like simple cells, the complex cell computation is replicated at a grid of locations *G_C_* (usually a subset of the simple cell grid *G_S_*) to produce a retinotopic channel of saliency response. Each channel is associated with a common feature transformation 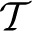, i.e., complex cells only combine the responses of simple cells of common transformation 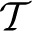. As illustrated in the left of Figure 1, the number of channels of complex cell response is identical to that of simple cell response.

### 2.2. Saliency detector implementations

The saliency measure of (5) can be implemented in three different ways, which are of interest for different applications of the saliency model.

#### 2.2.1. Biologically plausible implementation

**Figure 2:**
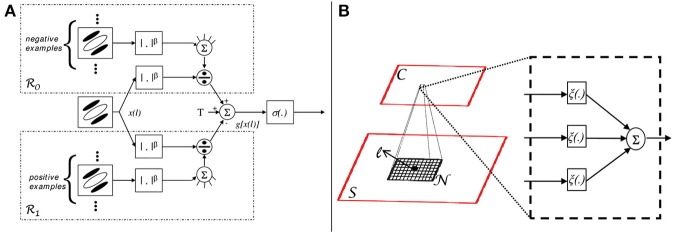
**Discriminant saliency computations**. **(A)** Simple cell (S unit). A unit of receptive field centered at location *l* computes a feature response *x*(*l*). This is then rectified, differentially divisively normalized by feature responses from areas *R*_0_ and *R*_1_, and fed to a sigmoid. The responses from the two areas act as training sets for the binary classification of *x*(*l*). More precisely, responses in *R*_0_ (*R*_1_) act as training examples for the negative (positive) class. The output *g*[*x*(*l*)] of the differential divisive normalization operator is the log-likelihood ration for the classification of *x*(*l*) with respect to the two classes (under the assumption of GGD statistics), as in (9). The sigmoid finally transforms this ratio into the posterior probability of the positive class, as in (3). **(B)** Complex cell (C unit). The bottom plane symbolizes the output of a layer of S-units, the top one the output of a layer of C-units. S-unit responses within a neighborhood *N*(*l*) are passed through non-linearity ξ(*x*) and pooled additively, to produce the response of a C unit. This implements the saliency measure of (8).

The saliency computations can be mapped into a network that replicates the standard neurophysiological model of visual cortex (Carandini et al., 2005). In biology, rather than the static analysis of a single image, recognition is usually combined with object tracking or some other dynamic visual process. In this case, saliency is not strictly a feedforward computation. In particular, the training sets *R_i_* of (2), used to learn the GGD parameters of a cell, are composed by responses of other cells, i.e., the target and background classes are defined by the lateral connections of a simple cell. An implementation of object tracking, by continuously adaptive recognition of the objects to track, using this type of mechanism is presented in Mahadevan and Vasconcelos (2013). In this implementation, the lateral connections are organized in a center surround manner, defining (1) the target class as the visual stimulus in a window containing the object to track and (2) the background class as the stimulus in a surrounding window. Under this type of implementation, a simple cell computes the LLR *g*[*x*(*l*)] by combining (4) and (2) into the divisive normalization operation

(9) $$g[x(l)]=\frac{|x(l){|}^{\beta }}{\frac{1}{\kappa }{\sum {}_{j\in R_{0}}\left|x(j)\right|}^{\beta }+\nu }-\frac{|x(l){|}^{\beta }}{\frac{1}{\kappa }{\sum {}_{j\in R_{1}}\left|x(j)\right|}^{\beta }+\nu }+T,$$

characteristic of simple cell computations (Heeger, 1992; Carandini et al., 1997, 2005). The LLR is then transformed into the posterior probability of (3) by application of a sigmoid transformation to the divisively normalized responses. An illustration of the simple cell computations is given in Figure 2A. Complex cells then implement the computations of (8), as illustrated in Figure 2B. When equipped with these units, the network of Figure 1 has a one to one mapping with the standard neurophysiologic model of the visual cortex (Carandini et al., 2005).

#### 2.2.2. Neural network implementation

Neural networks are commonly used to solve the computer vision problem of object recognition. In this setting, network parameters are learned during a training stage, after which the network operates in a feedforward manner. For these type of applications, the GGD parameters of (4) can be learned from a training set, using (2), and kept constant during the recognition process. This allows the simplification of the saliency operations. Namely, by combining (7) and (4) it follows that

(10) $$S(l)=\frac{1}{N(l)}\sum_{j\in N(l)}{\left\lfloor \gamma |x(j){|}^{\beta }+T\right\rfloor }_{+}$$

where $\gamma =\left(\frac{1}{\alpha_{0}^{\beta }}-\frac{1}{\alpha_{1}^{\beta }}\right)$, and $T=\log\left(\frac{\alpha_{1}}{\alpha_{0}}\right)$. This can again be mapped to the two layer network of Figure 1, but simple cells now simply rectify feature responses, according to

(11) $$\psi (x)={\left\lfloor \gamma |x{|}^{\beta }-T\right\rfloor }_{+},$$

while complex cells perform a simple average pooling operation. The resulting network is similar to the stages of rectifier linear units (ReLU) that have recently become popular in the deep learning literature (Nair and Hinton, 2010; Krizhevsky et al., 2012). When compared to the ReLU computation, *f*(*x*) = ⌊*x*⌋_+_, the parametric rectifier of (11) replaces static rectification by an adaptive rectification, tuned to the scales α_*i*_ of the feature distributions under target and background hypotheses.

This adaptation is illustrated in the right side of Figure 1. When α_1_ = α_0_, target and null distributions are identical and ψ(*x*) = 0 for all *x*. Hence, non-informative features for target detection are totally inhibited. When α_1_ > α_0_, the target distribution has heavier tails than the null distribution, i.e., the feature is *present* in the target. In this case (blue curve), the rectifier enhances large responses and inhibits small ones, acting as a detector of feature presence. Conversely, the null hypothesis has heavier tails when α_1_ < α_0_, i.e., when the feature is *absent* from the target. In this case (red dashed curve), the rectifier enhances small responses and inhibits large ones, acting as a detector of feature absence. In summary, the rectification introduced by the simple cells of (11) varies with a measure of discrimination of the feature *X*, based on the parameters γ and *T*. In result, the cell responses adapt to the feature distributions under the two hypotheses, allowing simple cells to have very diverse responses for different features. This is beyond the reach of the conventional ReLU rectifier. The adaptive behavior of ψ(*x*) is also reminiscent of optimal rules for image denoising (Chang et al., 2000). Like these rules, it thresholds the feature response, exhibiting a dead-zone (region of zero output) which depends on the feature type. Note that this results from (8), which is the Bayes decision rule for classification of the response *x*(*l*) into target and background. Hence, ψ(*x*) can be seen as an optimal feature denoising operator for the detection of targets embedded in clutter. The dead-zone depends on the relative scales of target and background distribution, according to

(12) $$\begin{array}{l} |x{|}^{\beta }\leq T/\gamma \text{ when }\alpha_{1}>\alpha_{0}\\ |x{|}^{\beta }\geq T/\gamma \text{ when }\alpha_{1}<\alpha_{0}. \end{array}$$

#### 2.2.3. Algorithmic implementation

It is also possible to compute the discriminant saliency measure with an algorithm that has little resemblance to any biological computation but provides insight into the saliency score. This follows from rewriting (5) as

$$\begin{array}{l} S(l)\;=\sum_{i=0}^{1}E_{X(l)|Y(l)}\left({\left\lfloor g(X)\right\rfloor }_{+}|i\right)P_{Y(l)}(i)\\ \;=E_{X(l)|Y(l)}\left({\left\lfloor g(X)\right\rfloor }_{+}|1\right)P_{Y(l)}(1)\propto E_{X(l)|Y(l)}\left(g(X)|1\right)\\ \;=\int_{N(l)}P_{X|Y}(x|1)\log\frac{P_{X|Y}(x|1)}{P_{X|Y}(x|0)}dx, \end{array}$$

where we have used the fact that ⌊*g*(*x*(*l*))⌋_+_ = 0 whenever *Y*(*l*) = 0 and *g*(*x*(*l*)) ≥ 0 otherwise. Hence, the saliency score can be interpreted as the computation, over the neighborhood *N*(*l*), of the Kullback-Leibler (KL) divergence between the probability distributions of the feature responses under the target and background distributions. Since the KL divergence is a well-known measure of distance between probability distributions, this confirms the discriminant nature of the saliency measure. Using (4), the KL divergence can be written as

(13) $$S(l)\;\propto E_{X(l)|Y(l)}[|x{|}^{\beta }|1]\left(\frac{1}{\alpha_{0}^{\beta }}-\frac{1}{\alpha_{1}^{\beta }}\right)+T$$

(14) $$\propto \frac{\gamma }{\beta }\alpha_{1}^{\beta }(l)+T,$$

where α^β^_1_(*l*) is the scale parameter of a GGD distribution with the responses observed in *N*(*l*). This enables a very simple computation of the saliency measure, using the following procedure.

1. From the feature responses *x_i_*(*j*) in the neighborhood *N*(*l*) estimate α^β^_1_(*l*), using (2).
2. Use (14) with α^β^_1_(*l*) and the model parameters α^β^_*i*_ learned from the training samples *R_i_* to compute the saliency score *S*(*l*).

#### 2.2.4. Discussion on different implementations

The three implementations above are equivalent, in the sense that they produce similar results on a given saliency task. They are suitable for different applications of the saliency measure of (5). In general, any model of biological computation has several implementations. For example, the convolution *y*(*l*) of a visual stimulus *x*(*l*) with a linear filter *h*(*l*) can be computed in at least two ways: (1) the classical convolution formula

(15) $$y(l)=\sum_{k}x(k)h(k-l)$$

or (2) the response to the stimulus *x*(*l*) of a convolutional neural network layer (Fukushima, 1980; LeCun et al., 1998) of linear units with identical weights, derived from the filter *h*(*l*). In this case, each network unit computes the output *y*(*l*) for a particular value of *l*. We refer to the first as the mathematical implementation and to the second as the biological implementation. While any biologically plausible network has an equivalent mathematical implementation, it is generally not true that all mathematical formulas can be implemented with biological circuits. Even when this is possible, the implementation may occur at different levels of abstraction. In general, an algorithm is considered biologically plausible if it can be mapped to a realistic model of neural computations (mapping from neuron stimuli to responses). This does not mean that it actually simulates neurons at the molecular level. It should, however, be able to predict the behavior of the neuron in neuroscience experiments.

In the discussion above, the algorithmic implementation of Section 2.2.3 is a mathematical implementation of the proposed saliency measure. It does not explicitly define units or neurons and is most suitable for the implementation of the measure as a computer vision algorithm, in a standard sequential processor. On the other hand, because it does not make explicit the input-output relationship of any particular neuron, it is not of great interest as a model of neuroscience. The biologically plausible implementation of Section 2.2.1 has the reverse role. Because it is fully compliant with the standard neurophysiological model of the visual cortex (Carandini et al., 2005), it predicts a large set of non-linear neuron behaviors which this model has been documented to capture (Carandini and Heeger, 2011). It could, thus, be used to study the role of these behaviors in object recognition. On the other hand, because it explicitly implements the computations of each neuron, its implementation on a sequential processor is much slower than the mathematical implementation of Section 2.2.3. Hence, it makes little sense to adopt it if the goal is simply to produce an efficient computer vision system. Finally, the neural network implementation of Section 2.2.2 is somewhere in between. It is a more abstract implementation than that of Section 2.2.1, in the sense that it does not explicitly include operations like divisive normalization. This makes it faster to compute and establishes a connection to recent models in the deep learning literature (Krizhevsky et al., 2012), which have been shown to achieve impressive object recognition results. These models can also be efficiently implemented in a GPU computer architecture, but are much slower on a traditional processor. Since this implementation achieves the best trade-off between fidelity to the neural computations and speed, we adopt it in the remainder of the paper. In particular, a CPU-based implementation of the neural network of Section 2.2.2 was used in all experiments of Section 4.

### 2.3. Hierarchical discriminant saliency networks

A *hierarchical discriminant saliency network* (HDSN) is a neural network whose layers are implemented by the saliency detector of Figure 1.

#### 2.3.1. HDSN architecture

**Figure 3:**
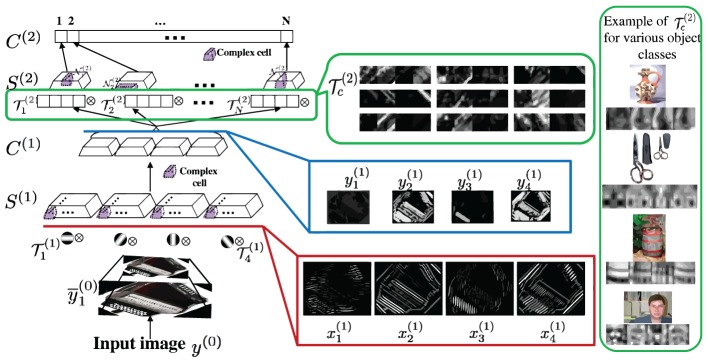
**Left:** HDSN with two layers. Each layer consists of a DSN, as in Figure 1. Layer *i* contains a sub-layer of simple (*S*^(*i*)^) and a sub-layer of complex (*C*^(*i*)^) units. The network has 4 channels in layer 1 and *N* in layer 2. Channel *c* is obtained by convolving the input of a layer with a template 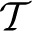
_*c*_, at several resolutions. Templates 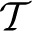
_^(1)^*c*_ of layer 1 are Gabor filters, templates 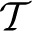
^(2)^_*c*_ of layer 2 are learned during training. **Center**: Gabor channels *x*^(1)^_*c*_ derived from the input image, corresponding saliency channels *y*^(1)^_*c*_ at the output of the first network layer, and example saliency templates 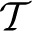
^(2)^_*c*_ learned by the second layer. **Right**: most discriminant template learned for each of four classes of Caltech101 (an example image is also shown for each class). Note that each template is composed of four image patches, derived from the four channels of the image representation in the first network layer.

The architecture of the HDSN is illustrated in Figure 3, for a two layer network. In general, a HDSN has *M* layers. As in Figure 1, layer *m* has two sub-layers: *S*^(*m*)^ of S units (simple cells) and *C*^(*m*)^ of C units (complex cells). S-units are located in a coordinate grid *G*^(*m*)^_*S*_, C-units in a coordinate grid *G*^(*m*)^_*C*_. Each sub-layer is organized into *C channels*. Channel *c* is based on the convolution of the layer input with a *template*, 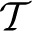
^(*m*)^_*c*_, shared by all its units. The processing of each channel is repeated at *R*^(*m*)^ image resolutions. The network of Figure 3, has *C*^(1)^ = 4 channels in layer 1 and *C*^(2)^ = *N* in layer 2.

Let *y*^(0)^ be the network input, and *y*^(*m*−1)^_*c*_ the output of *c*th channel of layer *m*−1. At layer *m*, *y*^(*m*−1)^ is first contrast normalized

(16) $${\bar{y}}_{c}(l)=\frac{y_{c}^{\left(m-1\right)}(l)}{\sum {}_{j\in Z(l)}\sum {}_{i}y_{i}^{\left(m-1\right)}(j)}$$

where *Z*(*l*) is a window, centered at *l*, with the size of template 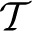
^(*m*)^_*c*_. The normalized input is then processed by the sub-layer of S-units, which first convolves it with the filters 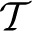
^(*m*)^_*c*_. This produces feature responses *x*^(*m*)^_*c*_(*l*), which are then sampled at S-unit locations *G*^(*m*)^_*s*_, and rectified by the parametric ReLU of (11),

(17) $$\psi_{c}^{\left(m\right)}\left(x\right)={\left\lfloor \gamma_{c}^{\left(m\right)}{\left|x\right|}^{\beta }-T_{c}^{\left(m\right)},\right\rfloor }_{+},$$

with parameters

(18) $$\gamma_{c}^{\left(m\right)}=\left(\frac{1}{{\left(\alpha_{c,0}^{\left(m\right)}\right)}^{\beta }}-\frac{1}{{\left(\alpha_{c,1}^{\left(m\right)}\right)}^{\beta }}\right)\;T_{c}^{\left(m\right)}=\log\frac{\alpha_{c,1}^{\left(m\right)}}{\alpha_{c,0}^{\left(m\right)}}.$$

The rectified filter responses are then fed to the sub-layer of C-units. Each C-unit computes the saliency score of (7) by simple averaging over its pooling window, i.e.,

(19) $$y_{c}^{\left(m\right)}(l^{\prime })=S_{c}^{\left(m\right)}(l^{\prime })=\frac{1}{\left|N^{\left(m\right)}(l^{\prime })\right|}\sum_{l\in N^{\left(m\right)}(l^{\prime })}\psi_{c}^{\left(m\right)}\left(x_{c}^{\left(m\right)}(l)\right)$$

The *c*th channel of this representation is the saliency map with respect to template 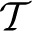
^(*m*)^_*c*_ and the *c*th channel of the output of layer *m*. The locations *l*′ are defined by the C-unit grid *G*^(*m*)^_*C*_. The pooling neighborhood *N*(*l*′) is usually smaller than the output of the afferent S sub-layer. Hence, both S and C-units have limited spatial support. However, *N*^(*m*)^(*l*′) can be location adaptive, i.e., depend on *l*′.

#### 2.3.2. Learning

The training of a HDSN consists of learning the templates 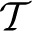
^(*m*)^_*c*_ and the GGD scales α^(*m*)^_*c*,0_, α^(*m*)^_*c*,1_ per layer *m*. Many approaches are possible to learn the templates 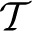
^(2)^_*c*_, including the backpropagation algorithm (LeCun et al., 1998), restricted Boltzmann machines (Hinton et al., 2006), clustering (Coates et al., 2011), multi-level sparse decompositions (Kavukcuoglu et al., 2010), etc. In this work, we adopt the simple procedure proposed for training the HMAX network in Serre et al. (2007); Mutch and Lowe (2008), where the templates 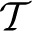
^(*m*)^_*c*_ of layer *m* are randomly sampled patches from the responses *y*^(*m*−1)^_*c*_ of layer *m* − 1, normalized to zero mean and unit norm. Given 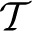
^(*m*)^_*c*_, the network is exposed to images from class *i* ∈ {0, 1}, and training samples *R*^(*m*)^_*c,i*_ collected. These consist of the responses *x*^(*m*)^_*c*_(*l*) across locations *l* and training images from class *i*. The scale parameters are then computed with (2).

#### 2.3.3. Object recognition

The HDSN is a hierarchical feature extractor, which maps the input image into a vector of responses of layer *C*^(*M*)^. For object recognition, this vector is fed to a linear classifier. In our implementation this is a support vector machine (SVM). The network topology is characterized by the parameters Θ^(*m*)^ = {*R*^(*m*)^, *G*^(*m*)^_*S*_, *G*^(*m*)^_*C*_, 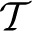
^(*m*)^, *N*^(*m*)^}, *m* ∈ {1, …, *M*}. As is usual in the hierarchical network literature, a good trade-off between object selectivity and invariance can be achieved by using (1) sparser grids *G*^(*m*)^_*S*_, *G*^(*m*)^_*C*_, (2) filters 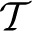
^(*m*)^ of larger spatial support, and (3) larger pooling neighborhoods *N*^(*m*)^, as *m* increases. This results in higher layer templates that are more selective for the target objects than those of the lower layers, without compromise of invariance. Since the selectivity-invariance trade-off of deep networks has been demonstrated by many prior works (Riesenhuber and Poggio, 1999; Serre et al., 2007; Krizhevsky et al., 2012), we do not discuss it here. In fact, the goal of this work was not to test the benefits of deep learning *per se*, which have now been amply demonstrated in the literature, but to investigate the benefits of augmenting the network with the saliency computations. Since, as we will see in the next section, many of the computer vision methods for object recognition can be mapped into two-layer networks, our study was limited to the network of Figure 3. This also had the advantage of enabling training from much smaller training sets.

In our implementation, *S*^(1)^ units use the 11 × 11 Gabor filters proposed in Mutch and Lowe (2008),

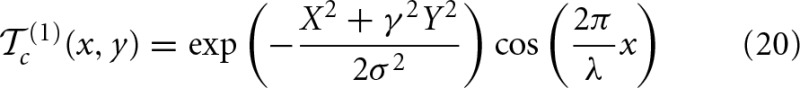



where *X* = *x* cos θ_*c*_ − *y* sin θ_*c*_, *Y* = *x* sin θ_*c*_ + *y* cos θ_*c*_, θ_*c*_ ∈ {0, π/4, π/2, 3π/4}, and γ, σ, and λ are set to 0.3, 4.5, and 5.6, respectively. This makes the first layer a detector of characteristic edges of the target. The training samples *R*^(1)^_*c,i*_ for learning the scale parameters α^(1)^_*c,i*_ are the set of Gabor responses *x*^(1)^_*c*_ to images of class *i* over the entire channel *c*. On the other hand, the templates of *S*^(2)^, 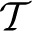
^(2)^_*c*_ = {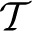
^(2)^_*c*,1_, …, 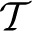
^(2)^_*c,C*^(1)^_}, span the *C*^(1)^ channels of the first layer, and are learned by random sampling, as discussed above. Since these templates are saliency patterns produced by layer 1 in response to the target, they are usually more complex features. The different complexity of the templates of the two layers warrants different pooling neighborhoods for C-units. Since simple features are homogeneous, layer 1 relies on a fixed neighborhood *N*^(1)^. On the other hand, to accommodate the diversity of its complex features, layer 2 uses template specific pooling neighborhoods *N*^(2)^_*c*_. Templates 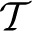
^(2)^ have dimension *n* × *n* × 4, for *n* ∈ {4, 8, 12, 16}, and are normalized to zero mean and unit norm (over the 4 channels). Pooling neighborhoods have area *S* ∈ {10, 20, 30%} of the size of layer 2 channels, and span *d* ∈ {3, 5, 7} scales. Like the templates, they are sampled randomly. These neighborhoods are also used to collect the training samples *R*^(2)^_*c,i*_ for learning the scale parameters associated with each of the templates. The network configuration is summarized in Table 1.

**Table 1:** **Configuration of the network used in all our experiments**.

	***R_(m)_***	***G^(m)^_S_***	***G^(m)^_C_***	***T^(m)^***	***N^(m)^***
*m* = 1	10 resolutions *r* ∈ {2^*i*/4^|*i* = 0, …, 9}	1 × 1 × 1	3 × 3 × 1 subsampling	Gabor filters of (20)	5 × 5 × 2 window
*m* = 2	same	1 × 1 × 1	Location and scale from which template is originally sampled	Randomly selected *n* × *n* × 4 templates, *n* ∈{4, 8, 12, 16}, with zero mean and unit norm	*S*% of image area and depth *d* in scale, where *S* ∈ {10, 20, 30}, *d* ∈ {3, 5, 7}

*Unless otherwise noted, n × m × l means a spatial step of *n* × m and a step of *l* across scales*.

**Figure 4:**
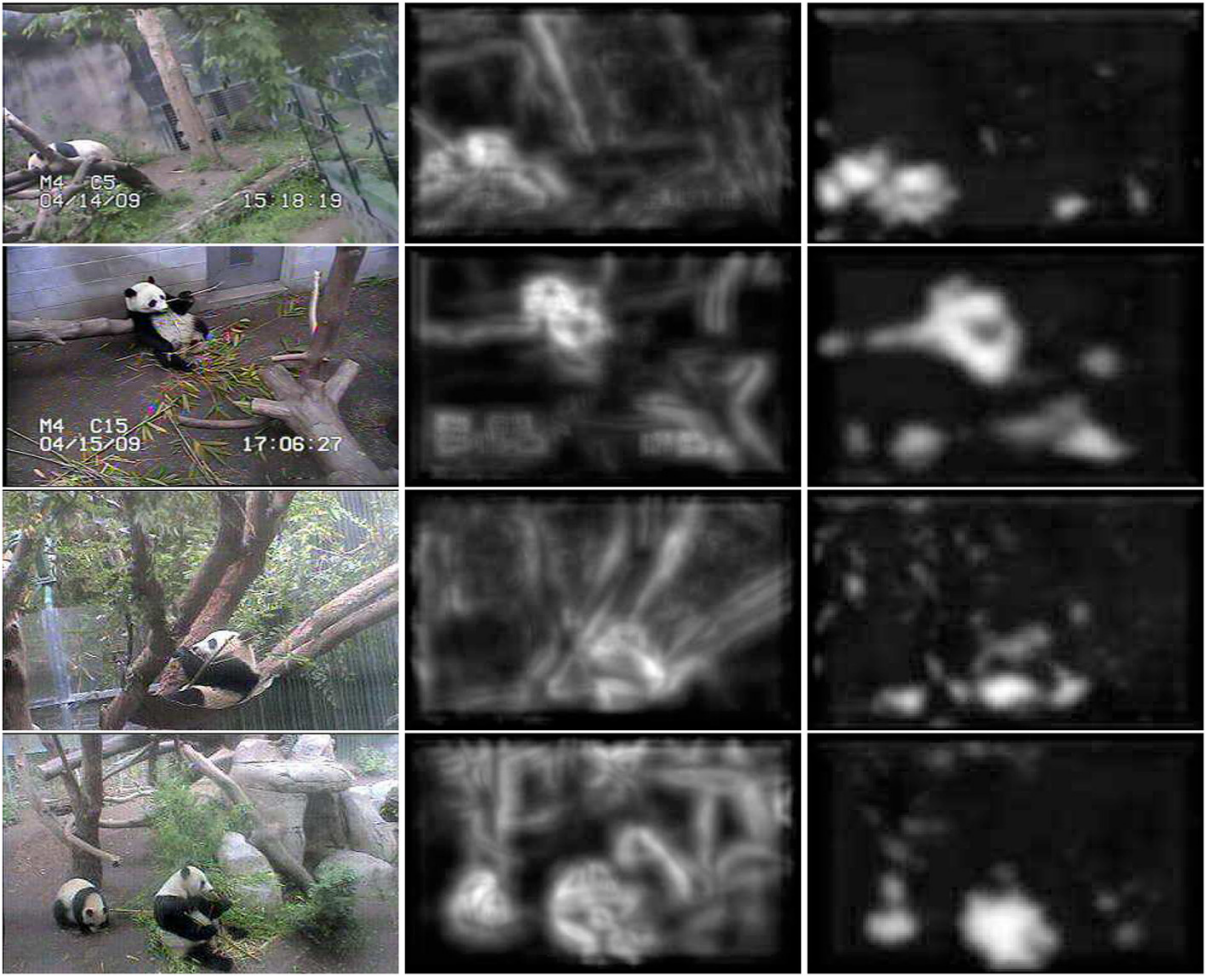
**Localization of panda bears in a complex environment**. **Left**: bear images. Note the highly variable pose of the bears and the strongly textured backgrounds. **Center**: saliency maps produced by a single layer HSDN. **Right**: saliency maps produced by a two-layer HDSN. The ability of the second network layer to learn discriminant saliency patterns reduces the number of false positives and enables significantly superior target localization.

Figure 3 illustrates the computations of the HDSN. It shows an image and the corresponding responses *x*^(1)^_*c*_ of the layer 1 Gabor filters, and *y*^(1)^_*c*_ of the layer 1 C-units. Note that, due to the class adaptive rectification of (17), the saliency responses *y*^(1)^_*c*_ amplify the filter responses *x*^(1)^_*c*_ of certain channels and inhibit the remaining. This allows the layer to produce a response that is more finely tuned to the discriminant features of the target class (in this example, the Caltech class “accordion”). Or, in other words, the layer highlights the features that are most distinctive of the target class. This, in turn, allows layer 2 to learn templates that are more discriminant of the target class than would be possible in the absence of the saliency computation. Note how the example templates 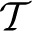
^(2)^ are selective for some of the feature channels. The inset on right of the figure presents the most discriminant template learned for four classes of Caltech101 (an example image of each of the classes is also shown). Note how the network has learned templates that are highly selective for the target objects. These templates are complex features (Vidal-Naquet and Ullman, 2003; Gao and Vasconcelos, 2005), which capture the spatial configuration of low-level features in target objects, resembling the receptive fields of cells in area IT (Riesenhuber and Poggio, 1999; Brincat and Connor, 2004; Yamane et al., 2008). Overall, while layer 1 processes edges, layer 2 captures shape information. When combined with the ability of the parametric ReLU rectifiers of (17) to behave as detectors of both feature presence and absence, this hierarchical learning of increasingly more selective templates enables the HDSN to compute saliency in challenging scenes. This is illustrated in Figure 4, using the pandaCam dataset, where background textures can be much more complex than the target object (panda bear). To be successful, the network must learn that the distinctive panda property is the absence of many of the features present in the background. The figure compares saliency maps produced by a HDSN with a single-layer (center column) and two layers (right column). Note how the latter produces saliency maps with less false positives and a much more precise localization of the target bears. The combination of (1) hierarchical learning of discriminant templates, and (2) detection of feature absence by parametric ReLUs, is critical for the network's effectiveness as a saliency detector.

## 3. Relationships to recognition models

In this section we compare HDSNs to previous object recognition models. We start by considering saliency models, then neural networks proposed for object recognition, and finally models from the computer vision literature.

### 3.1. Saliency models

Many stimulus driven, bottom-up, saliency models have been proposed in the literature. They implement center-surround operations (Itti et al., 1998; Gao and Vasconcelos, 2009), frequency analysis (Hou and Zhang, 2007; Guo et al., 2008), or detect stimuli with specific properties, e.g., low-probability (Rosenholtz, 1999; Bruce and Tsotsos, 2006; Zhang et al., 2008), high entropy (Kadir and Brady, 2001), or high complexity (Sebe and Lew, 2003). These models cannot account for the well-known fact that, beyond the stimulus, saliency is influenced by the task to be performed. For example, knowledge of target features increases the efficiency of visual search for a target among distractors (Tsotsos, 1991; Wolfe, 1998). This *top-down* component of saliency is classically modeled by modulating features responses (Treisman, 1985; Wolfe, 1994; Desimone and Duncan, 1995; Navalpakkam and Itti, 2007), i.e., global feature selection. This, however limits the ability to localize targets, since the selected filters respond to stimuli across the visual field. More recent top-down saliency models estimate distributions of feature response to target and background, and use them to derive optimal decision rules. These rules modulate feature responses spatially, according to the stimuli at different locations. A top-down saliency detector of this type is that of Elazary and Itti (2010). It differs from discriminant saliency through two simplifications: (1) assumption of Gaussian instead of generalized Gaussian responses (β = 2), and (2) use of the target log likelihood

(21) $$S^{\prime }(l)=\log P_{X_{c}^{\left(1\right)}|Y}(x_{c}^{\left(1\right)}(l)|1)$$

instead of (5), as saliency criterion (Elazary and Itti, 2010). In terms of the biological implementation discussed above, this corresponds to eliminating (1) C units, (2) the sigmoid σ(*x*), and (3) the top divisive normalization branch (see Figure 2A) of S units. We refer to such S units as target likelihood (TL) units, and the resulting network as likelihood saliency network (LSN).

### 3.2. Neural networks for recognition

HDSNs have commonalities with many neural network models proposed for object recognition.

#### 3.2.1. HMAX

Like the HDSN, the HMAX network follows the general architecture of Figure 3 (Serre et al., 2007). *S*^(1)^ units are Gabor filters, whose responses are pooled by *C*^(1)^ units, using a maximum operator

(22) $$y_{c}^{\left(1\right)}(l)=\underset{{}_{j\in N^{\left(1\right)}(l)}}{\max}x_{c}^{\left(1\right)}(j),$$

where we again denote filter responses by *x*^(*m*)^_*c*_(*l*) and pooling window by *N*^(*m*)^. The *S*^(2)^ sub-layer is a radial basis function (RBF) network with outputs

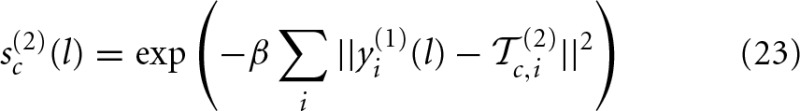



where β determines the sharpness of the RBF-unit tuning and 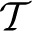
^(2)^_*c*_ is a template. Similarly to the proposed implementation of the HDSN, these templates are randomly selected during training, and have as many components 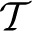
^(2)^_*c,i*_ as the number of layer 1 channels. *C*^(2)^ units are again max-pooling operators

(24) $$y_{c}^{\left(2\right)}(l)=\underset{j\in M^{\left(2\right)}}{\max}s_{c}^{\left(2\right)}(j),$$

where *M*^(2)^ is the whole visual field. A number of improvements to the HMAX architecture have been proposed in Mutch and Lowe (2008): a lateral inhibition that emulates divisive normalization, the restriction of *M*^(2)^ to template-specific neighborhoods [to increase localization of *C*^(2)^ units], a single set of templates shared by all object classes, and a support vector-machine (SVM)-based feature selection mechanism to select the most discriminant subset.

#### 3.2.2. Convolutional neural networks

Both the HDSN and the HMAX networks are members of the broader family of convolutional neural networks. These are again networks with the hierarchical structure of Figure 3, which date back to Fukushima's neocognitron (Fukushima, 1980). While early models lacked an explicit optimality criterion for training, convolutional networks trained by backpropagation became popular in the 1980s (LeCun et al., 1998). Classical models had no C units and their S units were composed uniquely of filtering and the sigmoid of (3). Recent extensions introduced S and C-like units per network layer (Pinto et al., 2008; Jarrett et al., 2009). While many variations are possible, modern S-units tend to include filtering, rectification, and contrast normalization. C-units then pool their responses. These extensions have significantly improved performance, sometimes producing staggering improvements. For example, Jarrett et al. (2009) reports that simply rectifying the output of each convolutional network unit drastically improves recognition accuracy. In fact, a network with random filters, but whose S-units include rectification and normalization, performs close to a network with extensively optimized filters. More recently, it has been shown that replacing the sigmoid of (3) by the ReLU nonlinearity *f*(*x*) = ⌊*x*⌋_+_ can significantly speed-up network training (Krizhevsky et al., 2012).

In this work, we consider in greater detail the network of Jarrett et al. (2009), which implements the most sophisticated S-units. The input of layer *m* is first convolved with a set of filters 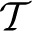
^(*m*)^_*c*_, producing feature responses *x*^(*m*)^_*c*_. These are then passed through a squashing non-linearity, absolute value rectification, subtractive, and divisive normalization, according to

(25) $$a_{c}^{\left(m\right)}(l)\;=|g_{c}\tanh x_{c}^{\left(m\right)}(l)|$$

(26) $$v_{c}^{\left(m\right)}(l)\;=\;a_{c}^{\left(m\right)}\left(l\right)-\sum_{c=1}^{C}\sum_{j\in M(l)}w(j)a_{c}^{\left(m\right)}(j)\;\sum_{j\in M(l)}w(j)=1/C$$

(27) $$u_{c}^{\left(m\right)}(l)\;=\frac{v_{c}^{\left(m\right)}(l)}{\max\left(\epsilon ,\sum_{c=1}^{C}\sum_{j\in M\left(l\right)}w\left(j\right){\left(v_{c}^{\left(m\right)}\right)}^{2}\left(j\right)\right)},$$

where *M*(*l*) is a 9 × 9 window. The normalized responses are finally fed to a layer of C-units, which implement spatial pooling

(28) $$y_{c}^{\left(m\right)}(l)=\sum_{j\in N(l)}u_{c}^{\left(m\right)}(j)$$

and subsampling. It is shown that unsupervised learning of the filters 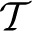
^(*m*)^_*c*_ is marginally better than adopting a random filter set, and relatively small gains result from global filter learning. More recently, Krizhevsky et al. (2012) have shown that state of the art results on large scale recognition problems can be obtained with a deep network, whose layers are slightly simpler than those of Jarrett et al. (2009). This is a network of five convolutional and three fully connected layers. Its convolutional stages consist of a sub-layer of S-units, which implement a sequence of filtering, divisive normalization with (27) and ReLU rectification, and a sub-layer of C-units, which implement the max pooling operation of (22). The filters 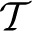
^(*m*)^_*c*_ are learned by back-propagation.

### 3.3. Computer vision models

Many object recognition methods have been proposed in the computer vision literature. Over the last decade, there has been a convergence to a canonical architecture, consisting of three stages: descriptor extraction, descriptor encoding, and classification. While the classification stage is usually a linear SVM, many of the recent object recognition methods differ on the details of the first two stages (Chatfield et al., 2011). We next show that this architecture can be mapped to the network of Figure 3.

#### 3.3.1. Canonical recognition architecture

**Figure 5:**
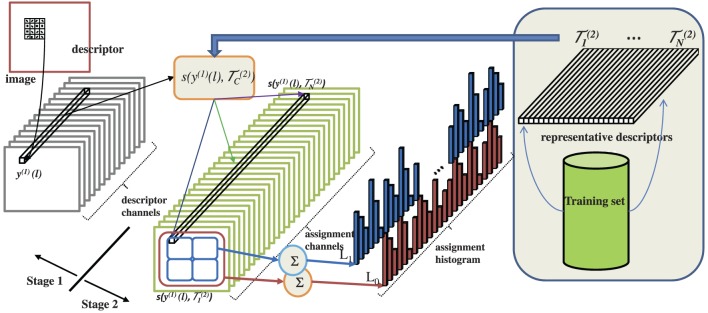
**Canonical architecture implemented by various popular object recognition methods**. Images are represented by sets of descriptors. A set of representative descriptors {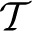
^(2)^_*c*_} is learned from an image training set. The descriptors *y*^(1)^(*l*) extracted from the image to classify are then encoded, with respect to this set of representatives. The encoding consists of assigning each descriptor to a subset of the representatives, using a similarity function *s*(*y*^(1)^(*l*), 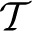
^(2)^_*c*_). This could be a probabilistic function, e.g., probability under a Gaussian mixture model, or a sparse encoding. The assignments are finally pooled spatially to produce assignment histograms, which are fed to a classifier, e.g., a support vector machine.

Figure 5 shows the two-stage canonical architecture for object recognition in computer vision. The first stage transforms an image into a collection of descriptors, usually denoted a bag-of-features. The descriptors *y*^(1)^(*l*) are calculated at image locations *l*, e.g., per pixel, in a regular pixel grid (dense sampling), or at keypoint locations (Lowe, 1999). We assume dense sampling, which produces best results (Zhang et al., 2007) and is more widely used. Descriptors are high-dimensional vectors, obtained by application of spatially localized operators at each image location. If each descriptor dimension *y*^(1)^_*c*_ is used to define a channel of this representation, descriptor channels can be interpreted as the channels of *C*^(1)^ output in Figure 3. The second stage computes an encoding of the descriptors extracted by the first. This is based on a set of descriptor templates, 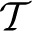
^(2)^_*c*_, learned from a training dataset. Descriptor templates can be the components of a model of the descriptor probability distribution, e.g., a Gaussian mixture model (GMM), kernel density, vector quantizer, or RBF network (Duda et al., 2001) or the basis functions of a sparse representation of descriptor space. When the former are used, we denote the encoding as probabilistic, while the term sparse encoding is used for the latter. Examples of probabilistic encodings include the minimum probability of error (MPE) architecture of Vasconcelos and Lippman (1997, 2000); Vasconcelos (2004a); Carneiro et al. (2007), the spatial pyramid matching kernel (SPMK) of Lazebnik et al. (2006), the naive-Bayes nearest neighbor (NBNN) classifier of Boiman et al. (2008), the hierarchical Gaussianization (HGMM) of Zhou et al. (2009), and many variants on these methods. Sparse encodings include, among others, the sparse SPMK method of Yang et al. (2009) and the locality-constrained linear (LLC) encoding of Wang et al. (2010).

The most popular encoding is probabilistic, namely a GMM with templates learned by either k-means or the expectation-maximization algorithm. In this case, the descriptor encoding reduces to computing a measure of descriptor-template similarity *s*(*y*^(1)^(*l*), 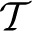
^(2)^_*c*_) and assigning the descriptor the closest template. It is also possible to rely on a soft assignment, where a descriptor is assigned to multiple templates with different weights. This is, for example, the case of sparse encodings. In all cases, the map of descriptor assignments to the *c*th template, 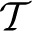
^(2)^_*c*_, is the *c*th channel of the stage 2 representation. Assignment channels are then pooled spatially, to produce the final image representation. For hard assignments, this is equivalent to representing the input image as a histogram of stage 2 assignments. The pooling operation can be performed over the entire image, sub-areas, or both. We next discuss how different computer vision methods map into this architecture.

#### 3.3.2. Stage 1: descriptors

Popular descriptors, e.g., SIFT (Lowe, 1999) or HoG (Dalal and Triggs, 2005), are measures of orientation dominance. While we discuss SIFT in detail, a similar analysis applies to others. The SIFT descriptor *y* ∈ ℝ^128^ is a set of 8-bin histograms of orientation response computed from intensity gradients. Location *l* contributes to histogram bin *k* with *a_k_*(*l*) = *r*(*l*)*g*(*l*)*b_k_*[θ(*l*)], where *r*(*l*), θ(*l*) are the gradient magnitude and orientation at *l*, *g*(*l*) a Gaussian that penalizes locations farther from the descriptor center, and *b_k_*(θ) a trilinear interpolator, based on the distance between θ and the orientation of bin *k*. The *k*th histogram entry is

(29) $$h_{k}=\sum_{l\in B}a_{k}(l),$$

where *B* is a 4 × 4 pixel cell. The descriptor concatenates histograms of 4 × 4 cells into a 128-dimensional vector, which is normalized, fed to a saturating nonlinearity τ(*x*) = max(*x*, 0.2) and normalized again to unit length. Using superscripts *q* ∈ {1, …, 16} for cells, and subscripts *k* ∈ {1, …, 8} for orientation bins, this is the sequence of computations

(30) $$\bar{h_{k}^{q}}=\tau \left[\frac{h_{k}^{q}}{\sum_{q,k}h_{k}^{q}}\right]=\tau \left[\sum_{l\in B^{q}}\frac{a_{k}(l)}{\sum_{q,k}\sum_{l\in B^{q}}a_{k}(l)}\right]$$

(31) $$s_{k}^{q}=\frac{\bar{h_{k}^{q}}}{\sum_{q,k}{\bar{h_{k}^{q}}}^{2}}\;y={\left(s^{1},\ldots ,s^{16}\right)}^{T}.$$

Note that (31) is a combination of divisive normalization (of *a_k_*(*l*) by responses in all cells *B^q^*), average pooling, and squashing non-linearity, similar to the sequence of (27) and (28). The main difference is the application of the non-linearity after pooling vs. after filtering, as in (25). (31) can be seen as pre-processing for stage 2, contrast normalizing stage 1 responses. This is identical to (16), the normalization of HDSN layer inputs. In summary, the SIFT computations can be mapped to a network layer similar to those discussed above.

In fact, the descriptor can be interpreted as a saliency measure, if *a_k_*(*l*) is replaced by the response magnitude |*x*^(1)^_*k*_(*l*)| of a Gabor filter with the *k*th orientation, a conceptually equivalent measure of oriented image energy. Defining

$$\begin{array}{l} \alpha =\sum_{q,j}\sum_{l\in B^{q}}|x_{j}^{\left(1\right)}(l)|=\sum_{q,l\in B^{q}}|x_{k}^{\left(1\right)}(l)|+\sum_{j\neq k}\sum_{q,l\in B^{q}}|x_{j}^{\left(1\right)}(l)|\\ \;=\sum_{q,l\in B^{q}}|x_{k}^{\left(1\right)}(l)|+\nu \end{array}$$

(31) reduces to *h^q^_k_* = τ[ϵ*^q^_k_*] where

(32) $$\epsilon_{k}^{q}=\sum_{l\in B^{q}}\frac{\left|x_{k}^{\left(1\right)}(l)\right|}{\alpha }$$

(33) $$\;\propto -\sum_{l\in B^{q}}\log P_{X_{k}^{\left(1\right)}}(x_{k}^{\left(1\right)}(l);\alpha ,1)$$

(34) $$\;\approx -\int_{B^{q}}P_{X_{k}}(x;\alpha^{q},1)\log P_{X_{k}}(x;\alpha ,1)dx$$

with *P_X_*(*x*; α, 1) as given in (1), and $\alpha^{q}=\sum_{l\in B^{q}}|x_{k}^{\left(1\right)}(l)|$. Hence, up to constants, ϵ*^q^_k_* is the cross-entropy between the responses of filter *X*^(1)^_*k*_ within cell *B^q^* and across the support of the descriptor. Assuming that the distributions are identical, this is the response entropy, a common saliency measure (Rosenholtz, 1999; Kadir and Brady, 2001; Bruce and Tsotsos, 2006; Zhang et al., 2008) that equates salient to rare (low-probability) events. Hence, SIFT can be interpreted as a saliency measure, which identifies as salient stimuli of rare orientation within a local image neighborhood.

#### 3.3.3. Stage 2: descriptor assignments

Under this interpretation, the templates 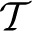
^(2)^_*c*_ are saliency templates^1^. For probabilistic models, the descriptor-to-template assignment of stage 2 is always a variation on layer 2 of the HMAX network. The likelihoods *s*^(2)^_*c*_(*l*) of the descriptor *y*^(1)^(*l*) under the components of a Gaussian mixture whose means are the templates 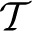
^(2)^_*c*_, *c* ∈ {1, …, *N*} are first computed with (23). These likelihoods are then mapped into posterior probabilities of component given descriptor, by a divisive normalization across channels

(35) $$p_{c}^{\left(2\right)}(l)=\frac{s_{c}^{\left(2\right)}(l)}{\sum_{c=1}^{N}s_{c}^{\left(2\right)}(l)}.$$

The RBF precision parameter β of (23) controls the softness of the assignments. When β → 0 the mixture model becomes a vector quantizer (Vasconcelos, 2004b) and *p*^(2)^_*c*_(*l*) = 1 for the template closest to *y*^(1)^(*l*), and zero for all others, i.e., assignments are hard. When β > 0 descriptors are assigned to multiple components, according to the posteriors *p*^(2)^_*c*_(*l*), i.e., assignments are soft. Some methods, e.g., MPE, HGMM, or NN, learn descriptor templates per object class and compute the posterior class probability

(36) $$P_{Y|X}(c|y^{\left(1\right)}(l))=\sum_{j\in I_{c}}p_{j}^{\left(2\right)}(l)$$

where *I_c_* is the set of indices of templates from class *c*. In summary, for probabilistic models, the second stage of the canonical architecture consists of the RBF network of HMAX plus the divisive normalization of (35), and can be complemented by (36). Overall, there are three types of layer 2 units: HMAX uses the likelihood units (LU) of (23), while the remaining approaches rely on the posterior units (PU) of (35), or the class-posterior units (CPU) of (36).

For sparse models, the assignments *p*^(2)^_*c*_(*l*) are obtained by minimizing a sparseness inducing assignment cost. For example, the assignments of SPMK are the solution of

(37) $$p^{\left(2\right)}(l)=\arg\underset{p}{\min}||y^{\left(1\right)}(l)-T^{\left(2\right)}p|{|}^{2}+\lambda ||p|{|}_{1}$$

where **T**^(2)^ is a dictionary with templates 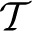
^(2)^_*c*_ as columns, ||*p*||_1_ the ℓ_1_ norm of *p*, and λ a regularization parameter. This produces a soft assignment, of sparsity (number of non-zero entries) controlled by λ. While sparse assignments can improve recognition performance, they have increased computational cost, since the optimization of (37) has to be repeated for each descriptor of the image to classify. This is frequently done with greedy optimization by matching pursuits (Mallat and Zhang, 1993), which involve multiple iterations over all templates in **T**^(2)^. We denote the units of sparse representation as *projection pursuit* (PP) units.

For both probabilistic and sparse models, the final step of stage 2 is an assignment histogram, computed by either average

(38) $$y_{c}^{\left(2\right)}(l)=\frac{1}{\left|N^{\left(2\right)}(l)\right|}\sum_{m\in N^{\left(2\right)}(l)}p_{c}^{\left(2\right)}(m),$$

or maximum

(39) $$y_{c}^{\left(2\right)}(l)=\underset{m\in N^{\left(2\right)}(l)}{\max}p_{c}^{\left(2\right)}(m),$$

pooling. The neighborhood *N*^(2)^(*l*) can be the entire image, in which case there are as many pooling units as descriptor templates, i.e., *N*, but is usually repeated for a number of subregions, using the pyramid structure introduced by SPMK and shown in Figure 5. This is usually a three-layer pyramid, containing the full image at level 0, and its partition into 2 × 2, and 4 × 4 equal sized cells at levels 1 and 2, respectively. In this case, there are a total of 21*N* pooling units.

### 3.4. Discussion

Table 2 summarizes the operations of various popular recognition methods. The table is organized by the type of saliency (none, bottom-up, or top-down) implemented by each of the methods. It should be noted that the template learning procedures are not necessarily tied to the network architecture. For example, HMAX could use k-means, and SPMK could use codebooks of randomly collected examples. In fact, many alternative methods have been proposed for codebook learning (Sivic and Zisserman, 2003; Csurka et al., 2004; Fei-Fei and Perona, 2005; Winn et al., 2005; Moosmann et al., 2007) or sparse representation (Mairal et al., 2008; Wang et al., 2010). It is, nevertheless, clear that the different methods perform similar sequences of operations. In all cases, these operations can be mapped into the network architecture of Figure 1 and implement at least some aspects of the standard neurophysiologic model (Carandini et al., 2005). However, the basic operations can differ in substantive details, such as the types of non-linearities, the order in which they are applied, etc. Since any combinations are in principle possible, the space of possible object recognition networks is combinatorial. This is amplified by the combinatorial possibilities for the number of parameters of any particular network configuration, e.g., receptive field sizes, subsampling factors, size of pooling regions, normalizing connections, etc. In result, it is nearly impossible to search for the best configuration for any particular recognition problem.

**Table 2:** **Mapping of various popular recognition algorithms to the canonical architecture of Figure 5**.

**Method**	**Stage 1**		**Stage 2**			
	**Units**	**Saliency**	**Templates**	**Units**	**Assignment**	**Pooling**
HMAX	Filter responses	–	Random	LU	Soft	Max
MPE	Filter responses	–	GMM	CPU	Soft	Sum
NBNN	SIFT	Bottom-up	Training set	CPU	Hard	Sum
SPMK	SIFT	Bottom-up	Codebook	PU	Hard	Sum
HGMM	SIFT	Bottom-up	GMM	CPU	soft	sum
Sparse SPMK	SIFT	Bottom-up	Sparse dictionary	PP	Soft	Max
LSN	SL	Top-down	–			
HDSN	DS	Top-down	Random	DS	–	Sum

*HMAX: (Serre et al., 2007), MPE: (Carneiro et al., 2007), NBNN: (Boiman et al., 2008), SPMK: (Lazebnik et al., 2006), HGMM: (Zhou et al., 2009), sparse SPMK: (Yang et al., 2009), LSN: (Elazary and Itti, 2010)*.

From a theoretical point of view, the main benefit of the HDSN is the statistical interpretation (e.g., computation of target probabilities) and functional justification (e.g., saliency detection) that it provides for all network computations. This results in clear guidelines for the sequence of network operations to be implemented, namely the S and C-units of Figure 2, clear semantics for normalizing connections (training feature responses under the target and background classes), and an abstract characterization of the unit computations, as in the algorithmic implementation of Section 2.2.3. It is thus possible to design network architectures for specific tasks, without the need for exhaustive search. In fact, the statistical nature of the underlying computations could be used to expand network functionality, e.g., by resorting to model adaptation techniques (Saenko et al., 2010; Dixit et al., 2011; Kulis et al., 2011) in order to reduce training set sizes, or belief propagation to enable more sophisticated forms of statistical inference, such as Markov or conditional random fields (Geman and Geman, 1984; He et al., 2004). For object recognition, some form of model adaptation is already enabled by the divisive normalization connections of Figure 2A) or, equivalently, the scale parameters α_*i*_ of the target and background distributions. As mentioned in Section 2.2.2, these enable the interpretation of S-units as the *parametric* rectification units ψ(*x*) of (11), which support a much richer set of network behaviors (e.g., sensitivity to feature absence) than commonly used non-linearities (such as the sigmoid or ReLU operations). By changing its scale parameters, the network can *adapt* to new recognition tasks without having to relearn new filters. This adaptation is also quite simple: it reduces to collecting samples of filter response to the target classes of interest and using (2) to estimate the scales α_*i*_. None of the other networks (or even computer vision algorithms) discussed above has this property.

Of all the recognition architectures discussed above, the HDSN is also unique in its explicit modeling of discriminant saliency, based on statistical modeling of the target and background distributions. In most other models, the saliency computation does not even involve the notions of target and background class, and the GGD scale is simply estimated from a neighborhood of the image to classify, as in (27) or (32). This strictly bottom-up definition of saliency cannot be tuned for recognition. On the other hand, the saliency maps of the HDSN identify feature responses discriminant for target detection, with all the advantages previously discussed: optimal feature denoising, modulation of saliency responses by the discriminant power of the underlying features, and ability to detect both feature presence and absence. These differences in turn have a non-trivial impact in the saliency templates 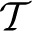
^(2)^_*c*_ of stage 2. SIFT templates are usually much less discriminant than those of Figure 3. By implementing saliency in layer 2, the HDSN complements this advantage with the identification of saliency configurations discriminant for target recognition. We next show that these properties make the HDSN more *efficient* in terms of image representation than all other models, achieving higher accuracies with fewer layer 2 units and a fairly simple training procedure.

## 4. Results

An extensive set of experiments was conducted to evaluate HDSN performance on saliency, object recognition, and localization tasks. All experiments were performed on datasets available in the literature, including Caltech101 (C101) (Fei-Fei et al., 2005), 15 scenes (N15) (Lazebnik et al., 2006), ALOI (Geusebroek et al., 2005), and the pandaCam dataset of Han and Vasconcelos (2011). Details of these datasets are given in the Supplementary Material.

### 4.1. Object recognition experiments

We start with object recognition. While, as shown in Table 2 the different approaches can be mapped to a common network form, the standard configurations of the different methods disagree even in the most elementary parameters, e.g., number of layer 2 units. For example, SPMK usually relies on a dictionary of size 1024 and a pyramid of 21 pooling regions. While this should be compared to an HMAX model of 21*K* units, only 4*K* are usually adopted in the HMAX literature. Methods that learn a codebook per class increase the number of units by a few orders of magnitude. In the worst case of Boiman et al. (2008) (as many units as training examples), the layer 2 RBF has 10 million units. This lack of uniformity makes it difficult to compare the different approaches. To overcome this problem, we implemented all units discussed in the previous sections and used them to build networks that are otherwise identical, i.e., have the same configuration, use the same learning procedure, etc. We then compared network performance on C101 and N15. A first experiment measured the impact of each unit of Table 2 on recognition accuracy. This experiment used a relatively small network, with fixed (Gabor) templates in the bottom layer and randomly sampled (from the first layer responses) templates in the second layer. In a second experiment, we built a larger HDSN and compared its performance to the results reported for the various recognition algorithms in the literature. This was mostly a sanity check, to ensure that the HDSN could achieve the results reported for these methods, using the parameters with which they were proposed. It is assumed that these parameters were optimized to guarantee the best results per method, of the network components, but allowing an unbiased estimate of the best possible performance per architecture.

#### 4.1.1. Impact of network units on recognition performance

To test the impact of network units on recognition accuracy, we started from a base network with the configuration of Table 1 and the following operations:

1. Local normalization of image intensities, according to (16);
2. *S*^(1)^ *units:* Gabor filters, no saliency;
3. *C*^(1)^ *units:* average pooling;
4. *S*^(2)^ *units:* 40 LUs with randomly selected templates 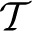
^(2)^_*c*_ per class, for a total of *C*^(2)^ = 600 channels for N15 and *C*^(2)^ = 4040 channels for C101.
5. *C*^(2)^ *units:* average pooling.

In a first experiment, we compared the impact of layer 1 units on network performance. This was done, by replacing the *S*^(1)^ and *C*^(1)^ units with those on the left of Table 3. The same Gabor channels were used across settings, the convolutional network layer (CN) was implemented with (25)–(28), SIFT with (31)–(31), and discriminant saliency (DS) with (17) and (2). The pooling operator was that which performed best for each network. Note that the type II network is identical to HMAX (Serre et al., 2007), and the first layer of the networks of type III, IV, and V is, respectively, layer 1 of the convolutional network of Jarrett et al. (2009), the first stage (SIFT) of the computer vision methods of Lazebnik et al. (2006); Boiman et al. (2008); Yang et al. (2009); Zhou et al. (2009), and layer 1 of the HDSN.

**Table 3:** **Recognition accuracy of a 2-layer network with different units**.

**Type**	**Simple unit**	**Pooling**	**L2**	**N15**	**C101**	**Type**	**L1**	**L2**	**N15**	**C101**
I	Filter	–	LU	58.7 ± 0.3	40.5 ± 0.8	I	DS	CPU	67.4 ± 1	61 ± 0.8
II	Filter	Max	LU	65.6 ± 1.3	52.8 ± 1	II	DS	PU	68.1 ± 1	62.1 ± 1.1
III	CN	Max	LU	67.1 ± 1.5	58.8 ± 1.3	III	DS	LU	68.3 ± 0.6	64.2 ± 1.3
IV	SIFT	Average	LU	67.5 ± 0.6	62.8 ± 0.9	IV	DS	DS	80 ± 0.6	69.2 ± 1.3
V	DS	Average	LU	68.3 ± 0.6	64.2 ± 1.3	V	DS^*^	DS	82.2 ± 0.7	69.9 ± 1.7

Left: impact of layer 1 units on recognition performance. Starting from a network (type I) with Gabor filters and no pooling in layer 1 and LU units with average pooling in layer 2, several enhancements were added to layer 1. These consisted of convolutional network (CN), SIFT, or discriminant saliency (DS) units. Max and average pooling operators were also tested, results are reported for the most effective. Right: impact of layer 2 units on recognition performance. In all cases, layer 1 consists of DS units. Layer 2 is implemented with CPU, PU, LU, or DS units. DS

* *reports to an enhanced layer 1, including feature selection*.

The table supports several conclusions. First, pooling significantly enhances recognition performance, as all methods with C units substantially outperformed the type I network. This finding confirms the importance of the spatial invariance attributed to this operation, and of C units in general. However, we *did not* find an advantage for either average or max pooling. Second, the addition of divisive normalization across features (bottom-up orientation saliency) implemented by both the CN and SIFT layers, further improved recognition accuracy. The gains of this operation were particularly significant on C101. This can be explained by the fact that shape is a more important cue for recognition in C101 (an object database) than in N15 (a database of scenes). Since this type of divisive normalization enhances edges with a dominant orientation, it produces crisper layer 2 templates, which are more informative about object shape. This enables large gains in C101 (from 52.8 to 62.8% for SIFT) but is also beneficial on N15 (from 65.6 to 67.5%). Third, for both datasets, the performance of the SIFT layer was superior to that of the convolutional network layer. This suggests that the sequence of S-unit operations of (31)–(31) is more effective than that of (25)–(28), but it is difficult to ascertain why. Finally, DS units had the best overall performance. It is worth noting that, while the SIFT and CN layers perform normalization *both* within (spatially) and across channels, DS units only require within channel normalization. This enables independent channel processing, considerably simplifying the implementation of this network. In fact, the HDSN layer has very little computational overhead with respect to the HMAX layer of the type II network. As discussed in Section 2.2.2, the only difference is the addition of the parametric ReLU units of (11). On C101, this boosts recognition accuracy from 52.8 to 64.2%. Overall, the HDSN layer has the lowest complexity among the top performing networks (types III to V).

To test the impact of the configuration of layer 2, we used a network with a layer 1 of DS units. Besides likelihood units (LU), layer 2 was implemented with posterior units (PU), class-posterior units (CPU), and DS units. Since the number of layer 2 channels is drastically reduced when CPU units are used (from the number of templates to the number of classes, e.g., 600 to 15 in N15 and 4040 to 101 in C101), and this reduces the effectiveness of the SVM that follows the network, we tried alternative CPU configurations. Best results were obtained, in preliminary experiments, by weighing PU units according to the posterior class probability, i.e., multiplying (35) by (36). The resulting accuracies are summarized in the right of Table 3, network types I–IV. Interestingly, neither PU nor CPU improved the performance of LU. Unlike layer 1, cross-channel normalization did not show any benefits in the second layer. Again, DS units achieved the best performance, substantially improving the recognition of LUs (68.3 to 80% on N15 and 64.2 to 69.2% on C101). In summary, both the adoption of DS units and the hierarchical computation of saliency produced substantial recognition gains. Note that the HDSN (type IV of right column of Table 3) is a fairly simple extension of HMAX (type II of the left column), both conceptually (addition of saliency) and algorithmically [addition of the parametric ReLU units of (11)]. A comparison to the HMAX performance, or even to an HMAX network with a DSN in the first layer (type V, left column) shows very significant improvements: from 66–68 to 80% on N15 and 53–64 to 69% on C101.

#### 4.1.2. Large network

The previous experiments were based on a relatively small network. We next compared the performance of a larger HDSN to the results reported in the literature for the methods of Section 3.3. We note that, when compared to these approaches, the features implemented by the HDSN (Gabor filters and randomly selected saliency templates) are fairly simple. The published results for the other algorithms are frequently based on much more complex features and feature selection. Examples include independent component analysis (ICA) (Kanan and Cottrell, 2010), sparse decompositions (Yang et al., 2009), or very large sets of random features (Jarrett et al., 2009). Pinto et al. (2008) has shown that a single layer network with many channels can outperform hierarchical networks with few channels per layer. We considered a limited set of enhancements of this type. The filter pool of the first layer was first augmented with 63 discrete cosine transform (DCT) filters of size 8 × 8 (the DCT set minus the average-DC-filter). This is a proxy for the expansion of Jarrett et al. (2009), who showed that a set of random projections can outperform a Gabor decomposition. Feature selection was then implemented by pooling the saliency measure of (7) across the visual field, per feature *X*. The 4 channels of largest saliency were selected, maintaining the dimensionality of layer 1 identical to HMAX (Mutch and Lowe, 2008). The resulting recognition accuracy is shown as type V in the right of Table 3, where DS^*^ means “DS with feature selection.” The more elaborate feature set had gains of 2.2% in N15 and 0.7% in C101. No further extensions were considered.

Table 4 compares these results to the literature, where different methods have very different numbers of layer 2 units. These are also shown in the table, which we organized by network dimensionality. The left half reports to “small” networks (≈400 units for N15, 4000 for C101), the right to “large” (≈20 K for both). The HDSN performs well in both regimes. The most interesting observation is, however, its performance among small networks, where it is far superior to the next best methods (82 vs. 75% on N15, 70 vs. 66% on C101). In fact, in N15, the small version of HDSN outperforms the large versions of SPMK, NBNN, and sparse SPMK. In C101, it is only outperformed by the large versions of HGMM, and sparse SPMK. It should be pointed that these are not the best results reported on these datasets. Better performance can usually be obtained using SVM classifiers with non-linear kernels, which we have not considered in our implementation. For example, on C101, the accuracy of a 4000 unit SPMK classifier can be boosted to 74.4% by addition of a chi-square kernel (Chatfield et al., 2011). This is slightly superior to the results reported in Table 4 for the combination of a 20,200 HDSN with a linear SVM. In summary, the HDSN of 20,000 units has learned a high-dimensional embedding similar to that of the kernel-SVM, which has orders of magnitude higher implementation complexity. This is particularly impressive given the simplicity of the random sampling procedure used to learn the HDSN templates. Again, the comparison to an equivalent network with no saliency computation (HMAX) shows very large gains (from 56 to 70% recognition rate on C101 with 4000 units).

**Table 4:** **Comparison of a 2-layer HDSN to various methods from the literature, on the 15 scenes and Caltech101 Datasets**.

**Method**	**#Layer 2 units**		**Recognition rate**		**#Layer 2 units**		**Recognition rate**	
	N15	C101	N15	C101	N15	C101	N15	C101
SPMK(*L* = 0)	400	200	74.8 ± 0.3	41.2	–	–	–	–
SPMK (*L* = 2)	–	4200	–	64.6	8400	–	81.4 ± 0.5	–
kNN-SVM	–	3030	–	66.2 ± 0.5	–	–	–	–
V1 model	–	4000	–	42 ± 0.5	–	86,000	–	65
HMAX	–	4075	–	56	–	–	–	–
NBNN	–	–	–	–	–	10 M	–	70.4
Sparse SPMK	450	5120	75.3 ± 0.5	64.8 ± 0.7	21,504	21,504	80.28 ± 0.9	**73.2 ± 0.5**
convNN	–	4096	–	65.5	–	–	–	–
HGMM	–	–	–	–	46,080	310,272	85.2	73.1
HDSN	450	4040	**82 ± 0.5**	**70 ± 0.5**	22,500	20,200	**85.4 ± 0.3**	73.1 ± 0.6

*Results are presented for different numbers of layer 2 units, and grouped into small (left) and large (right) networks. The comparison includes SPMK (Lazebnik et al., 2006), kNN-SVM (Zhang et al., 2006), V1 model (Pinto et al., 2008), HMAX (Mutch and Lowe, 2008), NBNN (Boiman et al., 2008), sparse SPMK (Yang et al., 2009), convNN (Jarrett et al., 2009), and HGMM (Zhou et al., 2009)*.

### 4.2. Comparison to saliency measures

**Figure 6:**
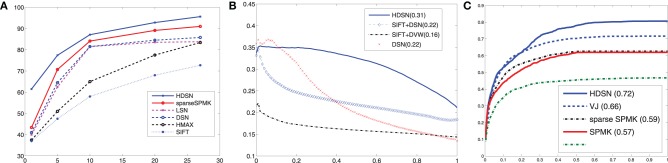
**(A)** classification accuracy vs. training set size on ALOI. **(B)** Precision-recall curves for object localization on pandaCam. **(C)** Detection rate vs. number of false positives per image for panda detection.

These results show that the HDSN outperforms architectures that use no saliency, or bottom-up saliency measures such as SIFT. The next comparison was to the top-down measure (LSN) of Elazary and Itti (2010). Since no software is available for this network, we compared the two approaches on ALOI, where LSN was originally evaluated. In addition to the HDSN (1000 units) and the methods evaluated in (Elazary and Itti, 2010)—LSN, HMAX (1000 units), and SIFT-based image matching (Lowe, 1999)—we also considered a single layer HDSN (denoted DSN) and sparse SPMK (1024 units). Figure 6A) compares the recognition rates of all methods, showing that the HDSN has the best performance. For example, with 27 training images per class, it has a recognition rate of 95.6%, while sparse SPMK achieves 91%, DSN 85.8%, LSN 83.8%, HMAX 83.4%, and SIFT 72.7%. These results confirm that both the addition of discriminant saliency (HDSN vs. HMAX) and its hierarchical computation (single-layer DSN vs. two-layer HDSN) lead to substantial gains in recognition performance.

### 4.3. Object localization and detection

**Figure 7:**
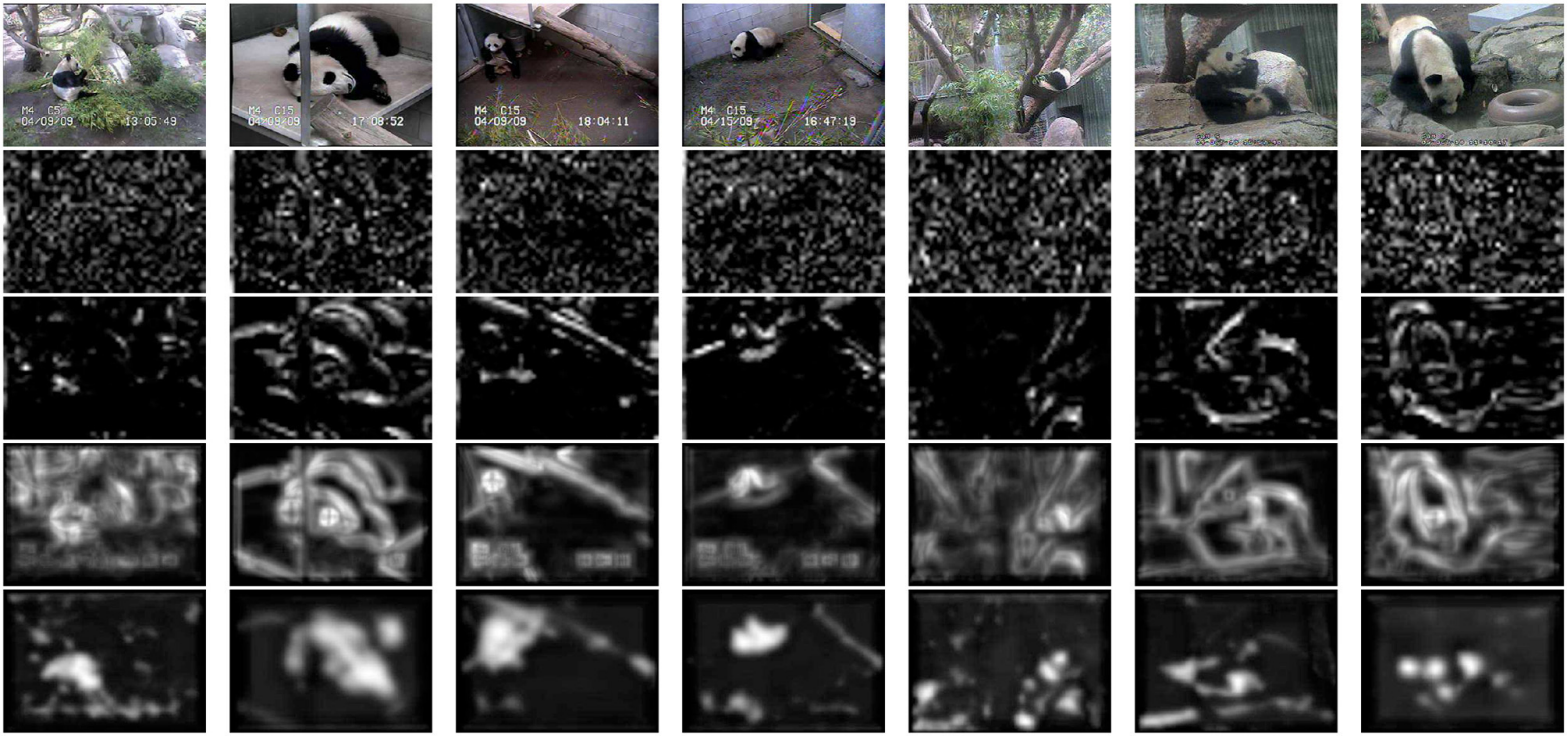
**Object localization on the pandaCam dataset**. **Top row**: example images. **Second row**: saliency maps produced by the combination of SIFT descriptors (layer 1) and discriminant visual words (layer 2). **Third row**: same for a combination of SIFT descriptors (layer 1) and discriminant saliency (layer 2). **Fourth row**: saliency maps produced by a single layer HDSN. **Fifth row**: same for a two-layer HDSN. In all cases, the saliency map is obtained by summing simple unit outputs across all channels.

We next considered the problem of object localization, on the pandaCam dataset, where we compared the performance of the HDSN to those of a saliency method based on SIFT in layer 1 and discriminant visual words in layer 2 (Dorko and Schmid, 2005) (SIFT+DVW), a HDSN with layer 1 replaced by SIFT units (SIFT+DS), and a single layer HDSN. SIFT+DVW is an intermediate between an RBF and a layer of DS units: it is based on visual words but emphasizes those that are discriminant for each class. Figure 7 shows saliency maps produced by the four methods, by simply summing the *S*^(2)^-unit responses across all feature channels. SIFT+DVW produces very noisy maps, with many false positives on the background, and few strong responses at target locations. The replacement of the DVW by the DS layer (SIFT+DS) suppresses most of this noise, but mostly produces edge maps, illustrating the limitations of SIFT: detection of simple features, failure to respond to the object interior, and poor selectivity for the target. While improving on DVW, the use of DS units in layer 2 cannot compensate for all these limitations. In fact, the single-layer HDSN produces better saliency maps than SIFT+DSN. Its maps are more selective for the target, have greater response toward the object interior, and respond more strongly to complex features such as the panda face. Finally, HDSN achieves the best performance, with saliency maps that are active in the target interior and have few false positives. These observations are confirmed by the precision recall curves of Figure 6B). The average precision is 0.31 for HDSN, 0.22 for single layer HDSN, 0.22 for SIFT+DS, and 0.16 for SIFT+DVW.

A final set of experiments was performed on object detection. An object detector was implemented by applying a box filter and non-maximum suppression to the saliency map of an HDSN with 200 layer 2 units. This was compared to a 6 component part model (partModel) of Felzenszwalb et al. (2009), sparse SPMK, SPMK, and the Viola-Jones (VJ) detector (Viola and Jones, 2004). Sparse SPMK, SPMK, and VJ used a sliding window, with windows of seven scales, and step size of 10 pixels. Non-maximum suppression was implemented as in Felzenszwalb et al. (2009), and applied to all approaches. SPMK and sparse SPMK used a spatial pyramid of 2 levels, and a codebook of 1000 visual words. Curves of detection rate vs false positives per image (fppi) are shown in Figure 6C). The partModel was unable to model pandas with the finite set of poses available, achieving the worst performance. Both sparse SPMK and SPMK produced a significant improvement, with sparse SPMK achieving slightly better performance. Another performance boost was achieved with the VJ detector. Finally, HDSN had the overall best performance. The detection rates at 0.3 fppi were 71.5% for HDSN, 66% for VJ, 58.6% for sparse SPMK, 56.8% for SPMK, and 43.8% for the partModel.

## 5. Conclusions

In this work, we have investigated the evolutionary benefits of integrating attention and object recognition, by introducing a joint model, the HDSN, for saliency and recognition. HDSNs are networks whose layers implement top-down saliency detectors based on features of increasing selectivity and invariance. This is accomplished by (1) learning saliency templates of increasing complexity and (2) adopting pooling operators of increasing support, in higher network layers. It was shown that HDSNs are consistent with the standard neurophysiologic model of the visual cortex but have a precise computational justification, and a statistical interpretation for all network computations. This enables the statistical learning of all network parameters and the explicit optimization of the network for recognition. The learning of HDSN parameters requires very simple mechanisms and has minimal computational cost over previous models, such as HMAX or convolutional neural networks, that lack an explicit connection to saliency. When compared to these models, HDSNs have a more precise mapping to the cortical neurophysiology, and explicitly account for both target and background hypotheses in the computation of all network layers. This results in saliency templates that are highly selective for the object classes of interest. The HDSN also introduces a new type of non-linearity, the parametric ReLU, whose parameters can be tuned for the detection of object classes of interest. This enables a number of functional enhancements, including optimal feature denoising mechanisms for recognition, modulation of saliency responses by the discriminant power of the underlying features, and ability to detect both feature presence and absence. A detailed experimental evaluation has provided evidence for the advantages of all these functional enhancements, as well as for the class-specific tuning inherent to discriminant saliency, and the gains of saliency layers using templates of increasing complexity, target selectivity, and invariance. It was also shown that normalization across orientation channels does not necessarily benefit recognition. This is an interesting finding, which enables much simpler networks and justifies the known cortical organization into orientation selective hyper-columns. Perhaps more importantly, the experiments presented suggest that there are non-trivial benefits in integrating attention and recognition. While attention is frequently modeled as a pre-processor (selector of regions), e.g., the classical dichotomy between pre-attentive and attentive vision, HDSNs assume that recognition *is* a component of attention and vice-versa. This was shown to substantially improve performance in core attention tasks, such as object localization, and core recognition tasks, such as object detection. In fact, it was shown that a single network can perform effectively in the problems of object localization, recognition, and detection, by a simple rearrangement of how the saliency maps produced by the different templates are processed: in parallel for recognition, and additively for localization and detection.

### Conflict of interest statement

The authors declare that the research was conducted in the absence of any commercial or financial relationships that could be construed as a potential conflict of interest.

## Footnotes

^1^This is a terminology for descriptor templates alternative to *visual words* (Sivic and Zisserman, 2003; Csurka et al., 2004), *textons* (Leung and Malik, 2001), *visemes* (Ezzat and Poggio, 2000), or others used in the literature.
