# Supplementary material for: Object recognition with hierarchical discriminant saliency networks
Source: Front Comput Neurosci. 2014 Sep 9;8:109. doi: 10.3389/fncom.2014.00109 (PMC4158795; doi:10.3389/fncom.2014.00109)
Supplement: Supplementary file 1 [file DataSheet1.PDF]

Frontiers in COMPUTATIONAL NEUROSCIENCE

Research Article
6 August 2014

# *Supplementary Material*: Object recognition with hierarchical discriminant saliency networks

**Sunhyoung Han** [1,*] **and Nuno Vasconcelos** [2]

[1] *Analytics department, ID Analytics, San Diego, CA, USA*
[2] *Statistical and Visual Computing Lab, Electrical and Computer Engineering, University of California San Diego, La Jolla, CA, USA*

Correspondence*:
Sunhyoung Han
Analytics department, ID Analytics, San Diego, CA, USA, shan@idanalytics.com

## 1 DATASETS

A number of datasets were used in the different experiments reported in this work. These are mostly datasets that have been widely used in the literature. Caltech101 (C101) is a popular benchmark for object recognition. It contains 9, 146 images from 101 object classes. In all our experiments, it was used with the experimental protocol of (**Mutch and Lowe**, 2008): multiclass recognition, with 30 training images per class, and a maximum of 50 for test. The 15 scenes dataset (N15) addresses the problem of image classification. It contains 4, 485 images from 15 scene classes, with 200 to 400 images per class, and was used with the experimental protocol detailed in (**Lazebnik et al.**, 2006). 100 images per class were randomly selected for training, the rest used as test set. In all C101 and N15 experiments, recognition rates were averaged over 5 runs on independent train and test sets. A linear SVM was used to classify the vector of network outputs in all cases. This vector was sphered before classification, by normalizing the mean and variance of each dimension to zero and one, respectively (**Mutch and Lowe**, 2008). The Amsterdam library of object images (ALOI) was used by (**Elazary and Itti**, 2010) to evaluate the recognition performance of saliency methods, namely LSN. It contains 108, 000 images from 1, 000 object categories, under 12 illumination colors, 72 illumination directions, and 72 viewpoints. All experiments on this dataset followed the experimental setup of (**Elazary and Itti**, 2010), where recognition rates are presented as a function of the number of training images. Object localization and detection experiments were conducted on the pandaCam dataset of (**Han and Vasconcelos**, 2011). This was assembled from a video feed, by the San Diego Zoo, depicting real time activity of a panda family in a natural habitat that includes bamboo, trees, ponds, a small rock cave, and several other small structures. The dataset contains 5, 018 panda images and 2, 987 negative examples. The pandas appear in a wide variety of body poses and light conditions, different degrees of occlusion by other objects in the scene, and are shot from various cameras. A bounding box is provided as detection ground truth on the positive images. Object localization performance was measured with precision-recall curves. Each curve was produced by thresholding the saliency map at various amplitude levels, measuring the overlap between the above-threshold region and the bounding box ground truth, and averaging over test images. Object detection performance was evaluated with the PASCAL measure, which requires an overlap greater than 50detected area and ground truth. The dataset is unique in that the background clutter is much more structured than the objects to detect. Background trees, branches, rocks and leaves all have a rich combination of structure, shape, and texture. This is unlike the pandas, which are mostly textureless and lack shape-defining edges.

## REFERENCES

Elazary, L. and Itti, L. (2010), A bayesian model for efficient visual search and recognition, *Vision Research*, 50, 1338–1352

Han, S. and Vasconcelos, N. (2011), Biologically plausible detection of amorphous objects in the wild

Lazebnik, S., Schmid, C., and Ponce, J. (2006), Beyond bags of features: Spatial pyramid matching for recognizing natural scene categories, in IEEE Conference in Computer Vision and Pattern Recognition

Mutch, J. and Lowe, D. (2008), Object class recognition and localization using sparse features with limited receptive fields, *International Journal of Computer Vision*, 80, 45–57
